# Safety and feasibility of percutaneous retrograde coronary sinus delivery of autologous bone marrow mononuclear cell transplantation in patients with chronic refractory angina

**DOI:** 10.1186/1479-5876-9-183

**Published:** 2011-10-26

**Authors:** Jorge Tuma, Roberto Fernández-Viña, Antonio Carrasco, Jorge Castillo, Carlos Cruz, Alvaro Carrillo, Jose Ercilla, Carlos Yarleque, Jaime Cunza, Timothy D Henry, Amit N Patel

**Affiliations:** 1 Division of Interventional Cardiology and Regenerative Medicine, Clí nica Maisó n de Santé, Lima, Peru; 2 Centro Cardiovascular San Nicolás, Don Roberto Fernandez-Viñ a Foundation, San Nicolas, Argentina; 3 Instituto de Criopreservació n y Terapia Celular, Lima, Peru; 4 Abbott Northwestern Hospital, Minneapolis, MN, USA; 5 University of Utah, Salt Lake City, UT, USA

## Abstract

**Background:**

Chronic refractory angina is a challenging clinical problem with limited treatment options. The results of early cardiovascular stem cell trials using ABMMC have been promising but have utilized intracoronary or intramyocardial delivery. The goal of the study was to evaluate the safety and early efficacy of autologous bone marrow derived mononuclear cells (ABMMC) delivered via percutaneous retrograde coronary sinus perfusion (PRCSP) to treat chronic refractory angina (CRA).

**Methods:**

From May 2005 to October 2006, 14 patients, age 68 +/- 20 years old, with CRA and ischemic stress-induced myocardial segments assessed by SPECT received a median 8.19*10^8 ^± 4.3*10^8 ^mononuclear and 1.65*10^7 ^± 1.42*10^7 ^CD34^+ ^cells by PRCSP..

**Results:**

ABMMC delivery was successful in all patients with no arrhythmias, elevated cardiac enzymes or complications related to the delivery. All but one patient improved by at least one Canadian Cardiovascular Society class at 2 year follow-up compared to baseline (p < 0.001). The median baseline area of ischemic myocardium by SPECT of 38.2% was reduced to 26.5% at one year and 23.5% at two years (p = 0.001). The median rest left ventricular ejection fraction by SPECT at baseline was 31.2% and improved to 35.5% at 2 year follow up (p = 0.019).

**Conclusions:**

PRCSP should be considered as an alternative method of delivery for cell therapy with the ability to safely deliver large number of cells regardless of coronary anatomy, valvular disease or myocardial dysfunction. The clinical improvement in angina, myocardial perfusion and function in this phase 1 study is encouraging and needs to be confirmed in randomized placebo controlled trials.

## Background

An increasing number of patients with coronary artery disease remain symptomatic with disabling angina despite the optimal use of antianginal medications and percutaneous or surgical revascularization [1,2]. Therapeutic angiogenesis is an experimental strategy utilizing angiogenic proteins, gene therapy or stem cells for inducing neovascularization of chronically ischemic myocardium [3,4]. Currently, the majority of clinical studies investigating autologous bone marrow mononuclear cells (ABMMC) transplantation as a treatment for ischemic myocardium have been performed in patients with acute myocardial infarction using intracoronary delivery [5-11]. In contrast, in patients with refractory ischemia only a few trials have been published and all have used intramyocardial delivery [12-16]. Percutaneous retrograde coronary sinus perfusion (PRCSP) is a well-established technique for delivery of cardioplegia solution in cardiovascular surgery and for protection against myocardial ischemia in patients undergoing high risk percutaneous coronary intervention (PCI) [17-19]. Delivery by PRCSP has been shown to be a reasonable alternative to intracoronary and intramyocardial delivery in preclinical therapeutic angiogenesis models using both angiogenic proteins, gene therapy and stem cell therapy [20]. The technique has the potential advantages of safety delivering a larger number of cells with more homogenious delivery across the myocardium to patients with refractory angina despite the presence of severe underlying coronary artery disease, valvular disease or previous myocardial infarction which may complicate delivery by either intracoronary or intramyocardial approach. The aim of the present study was to evaluate the safety of ABMMC delivered into the ischemic myocardium via PRCSP in patients with chronic refractory angina (CRA).

## Methods

### Patients

Patients with Canadian Cardiovascular Society (CCS) class III-IV angina despite maximal medical or surgical therapy who were ineligible for further percutaneous or surgical revascularization (based on coronary anatomy) and who had evidence for reversible ischemia on an exercise single photon emission computed tomography (SPECT) were enrolled. A committee comprising two cardiovascular surgeons and two interventional cardiologists determined the ineligibility for percutaneous or surgical revascularization. Patients with acute myocardial infarction, percutaneous or surgical revascularization within six months of enrollment in the study, a history of malignant disease, severe renal dysfunction, or unexplained hematology or biochemical abnormalities were excluded. The local ethics committee at Clinica Maison de Sante and Centro Cardiovascular San Nicolá s approved the protocol and all patients gave informed consent. The review and analysis of the data was also approved by the IRB at the University of Utah.

### Study protocol

The baseline screening assessment of patients included clinical evaluation, electrocardiogram (ECG), laboratory evaluation (complete blood count, blood chemistry, erythrocyte sedimentation rate, creatine kinase, and troponin T serum levels). Patients kept a record of daily angina frequency for three weeks, and the severity of angina was graded according to the CCS class at baseline, 3, 12, and 24 months. Within two weeks prior to cell therapy, exercise capacity was evaluated using bicycle ergometry in conjunction with SPECT imaging to assess myocardial ischemia and left ventricular (LV) function.

### Periprocedural evaluation

Patients had complete blood count, creatine kinase levels and ECG performed immediately prior to and immediate after the procedure. Complete blood count, creatine kinase, and troponin T levels were also assessed eight hours post procedure with an ECG prior to discharge. All patients were monitored in the cardiac intensive care unit for 12 hours after the procedure and were discharged the following day. A transthoracic echocardiogram was performed prior to discharge to detect the presence of pericardial effusion. The clinical follow-up evaluations including the laboratory tests described above were performed 3, 12 and 24 months after the injection procedure. At 12 and 24 months of follow-up exercise bicycle testing, and gated SPECT were performed.

### Assessment of exercise capacity

All patients performed a symptom-limited bicycle exercise test with a 20-W starting load and 10-W increments per minute at baseline and at 12 and 24 months after the injection procedure. Antianginal medication was continued. The test points were angina pectoris, physical exhaustion, dyspnea, significant decrease in blood pressure (10 mmHg), or achievement of maximal age-related heart rate. A 12-lead ECG was recorded before, during, and after the test. The total exercise duration, maximal workload achieved in percentages (expected for age, gender, height, and weigh) at baseline was compared to 12 and 24 months after ABMMC delivery.

### Single-photon emission computed tomography

For the SPECT examination, a one-day rest-stress protocol was used. On the same day, images at rest were obtained 60 minutes after the ^99 m^Technetium Sestamibi injection using a double-head SPECT camera as previously described. (17,18) Using the bicycle exercise test, ^99 m^Technetium Sestamibi was injected intravenously at peak exercise (85% of the estimated heart rate) which was continued for 2 minutes after tracer injection and images were obtained 30 minutes after the stress test. Reconstruction yielded long-and short -axis projections perpendicular to the heart axis. The short-axis slices were displayed in polar map format.

### Bone marrow aspiration and isolation of mononuclear cells

A volume of 300 ml of bone marrow was harvested from the iliac crest under local anesthesia and placed in heparinized Hanks' balanced salt solution. ABMMC were isolated by Hess 6% density gradient centrifugation with a final suspension volume of 50 ml and a mean cell concentration of 8.19*10^8 ^± 4.3*10^8 ^mononuclear cells and 1.65*10^7 ^± 1.42*10^7 ^CD34+ cells.

### Delivery Procedure

**Figure 1:**
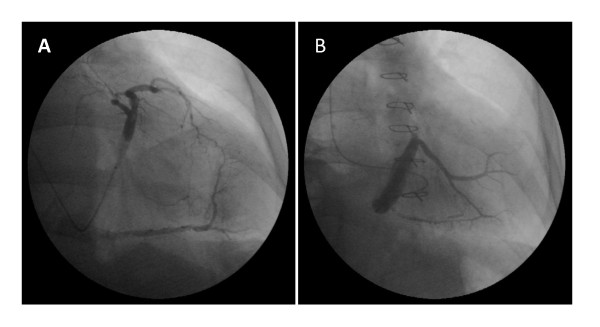
**A: Unselected transplantation of BMC into the coronary veins, B: Selected transplantation of BMC into the lateral veins**.

The femoral vein was cannulated with a 7 French sheath, a 6 French catheter was placed in the coronary sinus and a 0.035 mm hydrophilic guide wire was placed in the interventricular or lateral vein followed by placement of a peripheral balloon into the mid portion of the coronary sinus to allow nonselective delivery of cells. (Cook Medical, Indiana, USA). The balloon was inflated at very low pressure (1 to 2 atm) for 10 minutes producing stagnation of the flow as previously described with infusion of the ABMMC.(18,19) 50 mls of ABMMC were injected manually through the balloon at a rate of 10 mls per minute. The average total procedure time for cell delivery was 30 minutes.(Figure 1)

### Statistical analysis

Data are reported as median ± SDs. Quantitative data were compared using a paired, 2 tailed Student's test. Categorical data were compared using the Wilconxon signed rank test, a p < 0.05 was considered significant.

## Results

The baseline clinical characteristics of the 14 enrolled RA patients are listed in Table 1. All patients had three vessel disease and were deemed ineligible for further revascularization by the screening committee of physicians. The patient's antianginal regimen was not changed during the 24 month follow-up.

### Safety assessment

There was no evidence of inflammation or myocardial injury based on periprocedural laboratory evaluations (maximum erythrocyte sedimentation rate 20 ± 12 mm, maximum creatine kinase 137 ± 68 U/L, and maximum troponin T 0.010 ± 0.019 ng/mL) Ventricular arrhythmias were not observed during cell delivery or hospitalization and no patient had post procedural pericardial effusion by two-dimensional echocardiography before discharge. One patient died four months following the procedure due to liver cirrhosis, decompensated heart failure and pulmonary hypertension.

**Table 1:** Baseline Clinical Demographics

Demographic	ABMMC* (N = 14)
Median age	68

Female/Male ratio	1/6

Systemic Hypertension - N (%)	14 (100%)

Hyperlipidemia - N (%)	12 (85.7%)

Diabetes Mellitus - N (%)	4 (28.6%)

Previous myocardial infarction - N (%)	14 (100%)

Previous percutaneous coronary intervention - N (%)	4 (28.6%)

Previous coronary artery bypass surgery - N (%)	9 (64.3%)

*ABMMC = autologous bone marrow mononuclear cells

### Clinical outcomes

**Figure 2:**
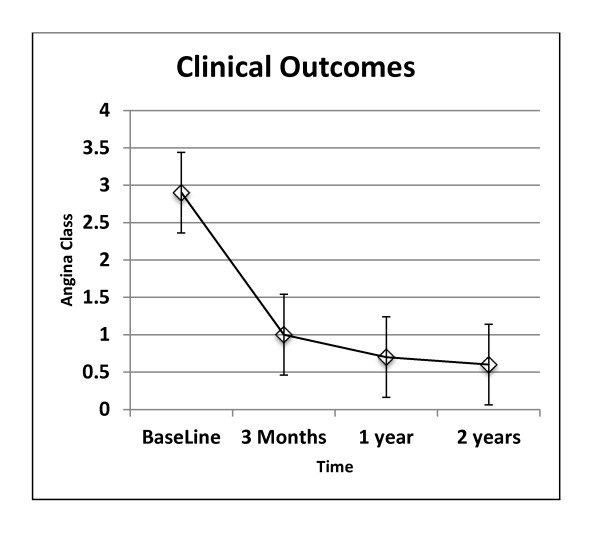
**Clinical outcomes of angina class**.

The frequency of angina episodes per day decreased from 2.9 ± 3.9 at baseline to 1.0 ± 1.4 at 3 months (p < 0.001), 0.7 ± 1.2 at 12 months (p < 0.001) and 0.6 ± 1.2 at 24 months (p < 0.001). Angina improved in 13/14 patients and the mean Canadian Cardiovascular Society class improved from 3.2 ± 0.6 baseline to 1.9 ± 0.7 at 1 year (p < 0.001) and 1.8 ± 0.4 at 2 years (p < 0.001). The individual changes for anginal episodes per day for the 14 patients are shown in Figure 2.

### Exercise capacity

**Figure 3:**
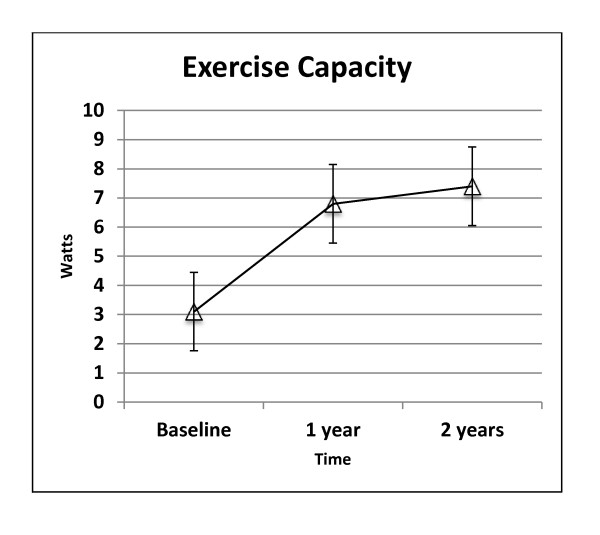
**Exercise Capacity**.

At baseline, 14 patients had exercise duration time of 3.1 ± 1.4 minutes. This improved to 6.8 ± 1.0 and 7.4 ± 1.7 minutes at 12 and 24 months of follow-up respectively, (p < 0.001). The individual changes for exercise time are shown in Figure 3.

### Myocardial perfusion by SPECT

**Figure 4:**
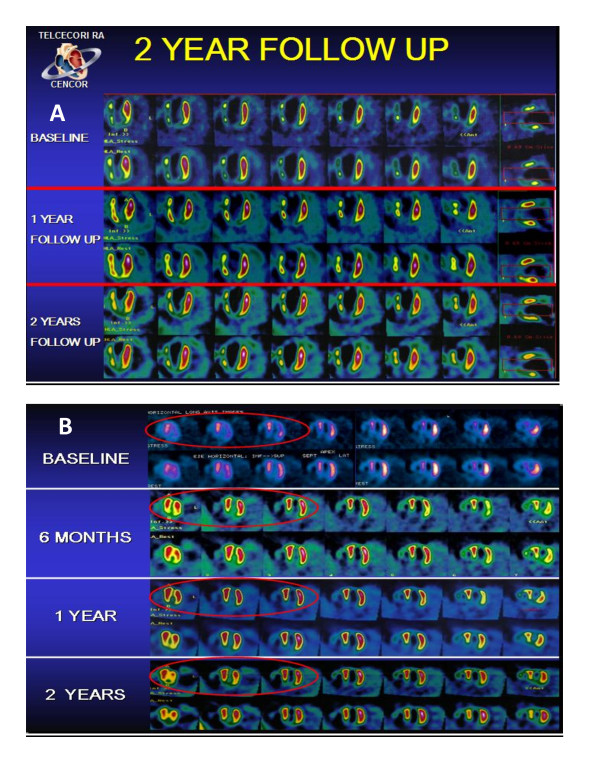
**A: Perfusion imaging in a patient at 2 year follow up, B: Perfusion imaging in another patient at 2 year follow up**.

The median percent of ischemic myocardium by SPECT was 38.2% at baseline and was reduced to 26.5% and 23.5% (p = 0.001) at 12 and 24 month follow-up (Table 2 and Figure 4). The number of segments with stress-inducible ischemia per patient improved 11.7% and 14.7% at 12 and 24 months of follow up. Examples of SPECT imaging of myocardial perfusion before and after ABMMC is shown for 2 patients in Figure 4A and 4B with improvements in both rest and stress perfusion.

**Table 2:** Analysis pre and post ABMMC transplantation: Canadian Cardiovascular Society class, rest left ventricular ejection faction and ischemic myocardium percent

	BASELINE (N = 14)	1 YEAR (N = 13)	2 YEARS (N = 13)	P value
REST Global LVEF* (%) Median	31.2	35.4	35.5	0.019

CCS† Median	3	2	2	<0.001

Ischemic Myocardium (%) Median	38.2	26.5	23.5	0.001

*LVEF = left ventricular ejection fraction; †CCS = Canadian Cardiovascular Society

### LV Function assessed by SPECT

**Figure 5:**
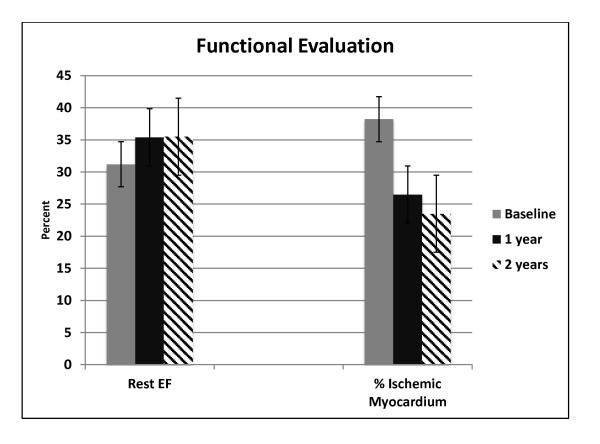
**Functional evaluation of myocardium**.

Median rest LV ejection fraction by SPECT at baseline was 31.2% and was improved to 35.4% and 35.5% at 12 and 24 month follow-up respectively (p < 0.019). (Table 2, Figure 5)

## Discussion

This study demonstrates that ABMMC delivered by PRCSP in patients with CRA and stress-inducible ischemia was safe, may reduce anginal symptoms and improve exercise capacity. Stem cell transplantation by PRCSP is an alternative route of delivery to the myocardium for patients with coronary artery occlusion at their initial portions, or for patients with severe multivessel disease or occluded venous and arterial grafts. In this study we have demonstrated that, in patients with chronic ischemia with or without severely impaired LV function, that ABMMC injection enhances myocardial perfusion. In addition we observed that improved systolic LV function occurred only in patients with low ejection at baseline. Improvement and maintenance of the LV ejection fraction at 24 months follow-up was most likely a result of increased myocardial perfusion and global regional wall motion. This resulted in a reduced LV end-systolic volume. Consequently, the therapeutic effect appears to be more related to enhanced myocardial contractility rather than induction of LV reverse remodeling. The findings of the present study are consistent with the hypothesis that ABMMC promote angiogenesis, resulting in increased myocardial perfusion [18]. The present study was not designed to assess the underlying cellular mechanism of bone marrow cell injection improving myocardial perfusion and LV function. Therefore, secretion of pro-angiogenic factors by the bone marrow cells and differentiation of bone marrow cells in endothelium cells, smooth muscle cells, or cardiomyocytes could have contributed to the described effect. The mechanism of angiogenesis could be caused by differentiation of bone marrow cells in endothelial and/or vascular smooth muscle cells or by the production of angiogenic cytokines, as previously proposed but has not been fully validated [19]. ABMMC by the PRCSP has been proposed as a novel therapeutic option for patients with coronary artery disease. Until now most clinical studies investigating ABMMC were performed in patients with acute myocardial infarction. Data from patients with chronic ischemia are scarce. At present, only few studies in patients with chronic coronary artery disease have been published [9-13]. The current results are in line with studies on the safety and feasibility of intramyocardial delivery of ABMMC in patients with chronic ischemia and angina pectoris. Tse et al ^9 ^described a reduction in anginal symptoms, improved wall motion and improved wall thickening at 3 'months follow-up in 8 patients. In addition, the area of hypoperfused myocardium was reduced on SPECT. Fuchs et al reported a reduction in anginal symptoms and improved myocardial perfusion in 27 patients with a trend toward improved LV ejection fraction [11]. Perin et al reported the same results in 21 patients [12]. In a randomized controlled study Losordo et al demonstrated data that CD34 + after GCSF can be safely transplanted via intramyocardial injection and may improve perfusion and reduce symptoms in patients with advanced coronary disease [14]. In this pilot trial ABMMC were successfully delivered in each patient without any major periprocedural events (i.e., death, myocardial infarction, ventricular arrhythmias, cardiac perforation, pericardial effusion or significant enzyme release) using PRCSP. The present study supports previously suggested beneficial effects in preclinical models of retrograde stem cells delivery for cardiac disease,[21,22] and the first to demonstrate improvement on symptoms, perfusion defects and LV function by PRVST in patients with preserved or decreased LV ejection fraction. However, we found similar improvements in patients with chronic ischemia with or without severely impaired LV function. This study suggests improvement and potential outcome durability of angina symptoms relief and better angina class, myocardial perfusion and contractility with this therapeutic approach in chronic refractory angina patients. We believe there may be clinical potential for this relatively novel method of cell delivery for patients suffering from refractory angina. We currently have a number of clinical trials using retrograde delivery of cells for both chronic refractory angina and heart failure based on this trial. Larger randomized trials will be needed to determine optimal cell numbers and to further understand the clinical outcomes.

## Abbreviation List

ABMMC: autologous bone marrow mononuclear cells; CCS: Canadian Cardiovascular Society; CRA: refractory angina; ECG: electrocardiogram; LV: left ventricular; PRCSP: percutaneous retrograde coronary sinus perfusion; SPECT: single photon emission computed tomography.

## Competing interests

The authors declare that they have no competing interests.

## Authors' contributions

JT, RFV, AC, JC, CC, AC, JE, CY, JC enrolled and treated the patients. JT, RFV, AP conceived the study. JT, TH, AP all help to draft the manuscript and were involved in the data analysis. All authors read and approved the final manuscript.
